# What are important areas where better technology would support women’s health? Findings from a priority setting partnership

**DOI:** 10.1186/s12905-023-02778-2

**Published:** 2023-12-13

**Authors:** Sharon Dixon, Sabrina Keating, Abigail McNiven, George Edwards, Philip Turner, Camilla Knox-Peebles, Neda Taghinejadi, Katy Vincent, Olivia James, Gail Hayward

**Affiliations:** 1 Nuffield Department of Primary Care Health Sciences, University of Oxford, Oxford, UK; 2 PPI advisor, CEO of Amref Health Africa UK, London, UK; 3 Department of Women’s and Reproductive Health, University of Oxford, Oxford, UK; 4 Exeter College, University of Oxford, Oxford, UK

**Keywords:** Femtech, Priority-setting partnership, Women's Health, Priority unmet needs

## Abstract

**Background:**

Women’s health has historically lacked investment in research and development. Technologies that enhance women’s health (‘FemTech’) could contribute to improving this. However, there has been little work to understand which priority unmet needs should be a focus for women’s health technology development. The voices of clinicians and those who experience and utilise these technologies (including those used at home or encountered in clinical settings) are needed to ensure that device development aligns with need, without risking exacerbating or creating health inequities.

**Method:**

We undertook a priority setting partnership project exploring unmet needs in women’s health and well-being where physical technologies or innovations could help. This comprised gathering feedback from: patients and clinicians using both qualitative surveys and discussions; collating and publishing these responses and asking for feedback; evidence checking unmet needs identified, and holding a partnership priority setting event to agree a top 10 and top 20 list of priorities.

**Results:**

We generated a ‘longlist’ of 54 suggestions for areas where better kit, devices or equipment could support women’s health. For three, we found evidence of existing technologies which mitigated against that need. We took the remaining 51 suggestions to a partnership priority setting meeting which brought together clinicians and service users. Through discussion as this group, we generated a list of the top 10 areas identified as priorities for technological development and improvement. These included better devices to manage examination, diagnosis and treatment of pelvic pain (including endometriosis), prolapse care, continence (treatment and prevention, related to pregnancy and beyond), menstruation, vaginal pain and vaginismus, point of care tests for common infections, and nipple care when breastfeeding.

**Conclusion:**

The top priorities suggest far-reaching areas of unmet need across women’s life course and across multiple domains of health and well-being, and opportunities where innovation in the devices that people use themselves or encounter in health settings could potentially enhance health and healthcare experiences.

**Supplementary Information:**

The online version contains supplementary material available at 10.1186/s12905-023-02778-2.

## Background

Women’s health has been historically under-represented in research [1, 2], resulting in a relative delay in medical knowledge and technological development. One response to this has been increased attention towards ‘FemTech’ – a term which has come to encompass the “services, products, and software designed to address the unique biological and medical needs of women” [3]. The 2022 Women’s Health Strategy for England, a policy imperative seeking to address the imbalance in women’s health experiences and outcomes, highlights the need to harness the potential of FemTech to “empower women to have fair access to clinically safe technologies – whether diagnostic, therapeutic or preventive – to ultimately improve health outcomes for women” [4].

However, concerns have been raised about a misalignment between the needs of women and the focus of industry, which may undermine the potential of FemTech to equitably improve women’s health [5]. Technology-based solutions have been criticised for focussing on users who are health-literate and socioeconomically privileged [5, 6], for not addressing the needs of marginalised groups [ 5, 6], and for lacking adequate data privacy [5–8]. Researchers have also argued that important women’s health issues are under-represented due to industry’s tendency to focus on users’ reproductive capabilities as opposed to adopting a broader life-course approach to women’s health [9, 10]. To date there have been limited attempts to establish what women and the clinicians who care for them consider the most important unmet needs for health technologies. In turn, this could result in missed opportunities to improve women’s health by focussing on technologies that match women’s own health priorities and better account for health inequities [11].

This project used a modified priority setting partnership (PSP) approach to generate a priority list of unmet needs for women’s healthcare technology, with a focus on physical technologies (devices, products, equipment) as opposed to mobile apps and digital products. We included people who use and deliver women’s healthcare when collecting, collating and prioritising suggestions for unmet needs for healthcare technology.

## Methods

The project team included GPs, social science researchers, a gynaecologist and a sexual and reproductive health doctor. The team were guided by a clinical stakeholder panel of five clinician advisers (including two GPs, an obstetric consultant, a physiotherapist, and a urogynaecology specialist) and a patient stakeholder panel including five patient and public involvement and engagement (PPIE) representatives with lived experience of women’s health problems.

Our methods and approach were inspired and guided by the James Lind Alliance approach to priority setting partnerships.

**Fig. 1:**
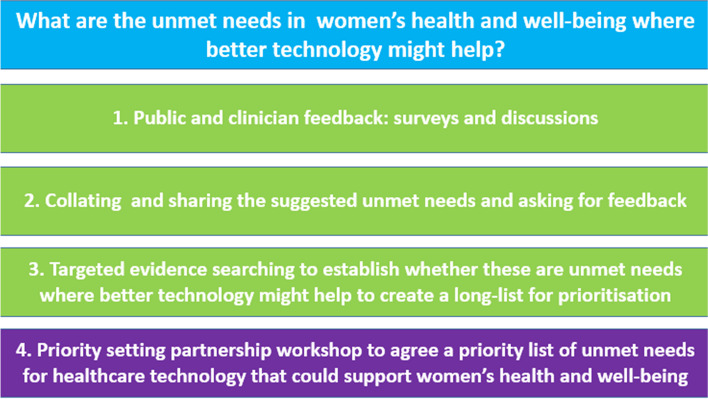
Schematic representation of project process

The project process is summarised in Fig. 1 and described below.

We report both the methods and then our project findings aligned within the project sequence of steps undertaken.

### Public and clinician feedback: surveys and discussions

#### Surveys

We designed brief anonymous qualitative surveys to ask members of the public and clinicians for their ideas about aspects of women’s health and well-being which could be improved by new or better ‘technology’, defined within this project as equipment, products, resources, kit, or devices. We asked for ideas about things that were currently problematic and for any suggestions about how these problems could be improved by better technology. The word ‘better’ was chosen with public and stakeholder input, reflecting the intention to consider ways that a technology could be easier to use, more broadly accessible in cost or availability, or more robustly evidence-based. We asked respondents to describe what the problem was, why it was a problem, and for any ideas about solutions. The surveys are included in Appendix A.

The surveys were developed and piloted with our stakeholder and patient panels, who also supported us in achieving wide dissemination of the surveys.

Surveys were shared through the project webpage and promoted via social media including Twitter. We shared the surveys with a wide range of relevant professional and advocacy organisations including RCOG Women’s Voices, the Primary Care Women’s Health Forum, the Royal College of Midwives, Cysters, Pregnant then Screwed, the NCT, Oxfordshire Maternity Voices Partnership, and NIHR PPIE networks.

#### Patient and public involvement and engagement (*PPIE) and healthcare professional discussions*

To ensure as wide as possible a range of views were included, we undertook discussion groups or one-on-one conversations with PPIE participants, aiming to learn about areas of unmet need in greater depth or to explore the opinions of women who might have been less likely to respond to our survey. We advertised widely for participants, seeking women with a range of background and experience (age, lived health experience, disability, ethnicity, socioeconomic status). We distributed a flyer asking for expressions of interest through patient and public involvement networks, community advocacy organisations, and on social media. Conversations were conducted remotely, at times and dates convenient for the PPIE participants, and as individuals or in groups aligned with participant preference.

We also offered small group or individual discussions with health professionals, to ensure key clinical voices were represented throughout our project, for example midwives. We distributed the flyer and information about our project through professional networks, including Primary care Women’s health networks, Community Sexual Health networks, and the Royal College of Midwives. Researchers made written summary notes following these conversations.

#### Collating and sharing unmet needs and asking for feedback

We collated all the unmet needs suggested in the surveys and individual and group discussions. This collated summary was made available on the project website.

We developed a survey tool asking for feedback and early guidance on prioritisation for the unmet needs detailed in the project longlist. The link to the ranking tool was promoted on social media, alongside the surveys, and remained open until the final project meeting. The collated summary is available here: (EMPOWER — NIHR Community Healthcare MIC) and in Appendix B, and the ranking survey is included as Appendix C.

#### Targeted evidence searching to establish unmet needs

We undertook targeted literature and evidence searches, and used expert guidance, to look for products, kit, devices and equipment which could meet the unmet needs on our longlist. We appraised the technology readiness level [12, 13] of potentially relevant technologies. We searched for evidence of clinical- or cost- effectiveness in scientific literature, but also looked for evidence of commercial availability alongside commercial evaluations and testimonies and searched in trial registries for ongoing and funded research, and in product development searches. Following our searches, we categorised the unmet needs as unmet, inadequately met, or met. The tool we used to appraise technology readiness and our search strategy approach is included within Appendix D.

The final list taken to the partnership meeting for prioritisation included all unmet, partially met, or inadequately met needs.

#### Priority setting partnership workshop

We held a hybrid online and in person collaborative priority setting meeting on May 10th, 2022. The aim was to agree a top 20 and top 10 set of unmet needs. At the workshop, we first asked attendees to work in small groups to rank all the final longlist unmet needs, with the aim of identifying the top 40 (or less) suggestions to be taken forward to the subsequent round of ranking. Three groups were held in person and two online. Each group included a mixture of health professionals and patient and public participants.

The groups came back together to represent their perspectives on which items should be taken forward to the next round. The rankings for each statement from each group were collated and added together. There was consensus agreement on the shortlist to take through to the second round of ranking. Participants were permitted to identify areas where there was potential to bring related suggestions together or to enhance suggestions to ensure they were most applicable. We reconvened as small groups to discuss the shortlisted suggestions prior to coming back together for the final consensus conversation at which we agreed a top 20 and then top 10 suggestions.

The top 20 and top ten suggestions were circulated to all participants after the meeting, asking for feedback and to check for clarity of understanding.

#### Ethical approvals

This is a patient and public involvement priority setting partnership project developing suggestions for research needs, rather than research per se. This project was conducted and reported in line with established PPIE guidance (NIHR Involve). The project and surveys were reviewed by the University of Oxford Medical Sciences ethical committee who advised that, because these were anonymous patient and public involvement and engagement questionnaires, formal ethical approval was not required; thus ethics approval and informed consent were deemed unnecessary and were waivered. Stakeholder conversations were not recorded or transcribed to ensure anonymity. This project was conducted in line with established James Lind Alliance methodology.

## Note on language

While the language of ‘women’s health’ has been used within this study, we acknowledge the overlapping and discrete health needs of non-binary and transgender individuals, and the necessity of technological development to attend to this. In the initial stages of project design, we consulted PPIE advisors with lived experience of accessing women’s health care who indicated the usefulness and clarity of using ‘woman forward’ language. This decision was also informed by the 2022 Women’s Health Strategy for England, which accounts for transgender and non-binary health needs as an integrated part of the nominal field of women’s health [14]. The project survey was open to all respondents, and we did not collect information about gender identity. For this reason, we have used gender neutral language when referring to participants or participant responses.

## Results

### Public and clinician feedback: surveys and discussions

We received 112 public and 45 clinician responses to our survey. Many responses included more than one idea, and in total this represented 235 patient suggestions and 94 clinician suggestions (total *N* = 329). There was good representation from public respondents across age and region of the UK. The age of respondents ranged from 18 to 88, with a median age of 42. Documented ethnicity on the questionnaire was: white British (70%), white other (7%), South Asian ethnicity (5%), any other ethnicity (3%) declined to respond (7%). Respondents were mostly from Southeast England (48%) and London (17%), but there was representation from the Midlands, Northwest and Northeast England, Yorkshire, Scotland and Wales.

Clinician roles included midwifery, nursing, obstetrics, gynaecology, primary care, community care, and physiotherapy. The majority worked in Southeast England (64%), Southwest England (11%), or London (9%), but there was representation from Northeast England, Northwest England, the Midlands, Yorkshire and Wales.

We conducted three individual and two online group conversations each including five participants. These included participants with lived experience of disability, and women from African and Asian backgrounds. We held individual and group conversations with community sexual and reproductive health clinicians (*N* = 5), GPs (*N* = 2), and midwives (N = 5).

### Collating and sharing the unmet needs and asking for feedback

In this project, our focus was on identifying members of the public and clinicians’ ideas about priorities for products, equipment, kits or devices that could enhance women’s health. We shared the list of all suggestions on our website and asked for feedback through the surveys and online ranking tool. All of the suggestions prioritised through the ranking tool were taken forward to the final meeting.

### Targeted evidence searching to establish whether these are unmet needs

After targeted evidence searching, three of the 54 suggested unmet needs were deemed to be met, on the basis of existing evidence or completed or ongoing large scale trials which were evaluating technologies that could meet these needs. These three suggestions were: home blood pressure monitoring in pregnancy; self-taken swab tests for cervical screening; and breast cancer screening that did not involve uncomfortable breast compression. Please see Appendix E for a summary of these searches. For the remaining 51, we adopted a broad approach to inclusion, taking forward any needs where there was considered scope to improve development.

### Priority setting partnership final meeting

This meeting included 28 participants (20 in person, 8 online) alongside the project team, who were also present at the meeting. This represented 16 public and patient participants (with varied women’s health experiences and a range of ages, ethnicities and disability) and 12 clinicians (including obstetrics, midwifery, gynaecology, primary care, contraception and sexual health).

**Fig. 2:**
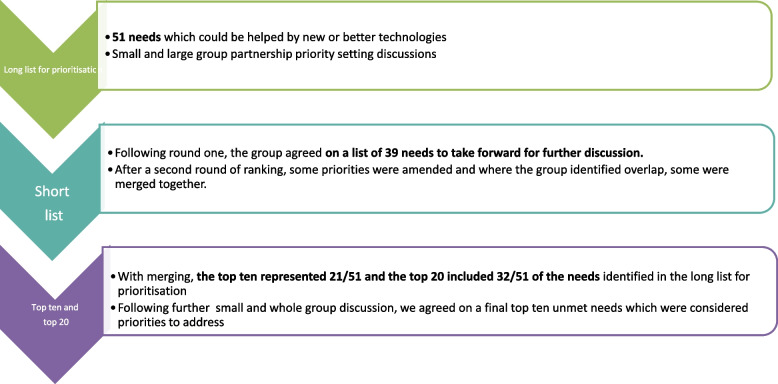
Schematic representation of processes at the final priority setting meeting

The final longlist for prioritisation included 51 unmet needs which were taken to the priority setting partnership final meeting. The process during the meeting is summarised in Fig. 2.

The meeting used a consensus approach throughout. We discussed each item in small groups, using printed cards in person and online jam board tools for the virtual groups. This approach sought to give time and space for each suggestion and voice to each participant. We did not specify how groups should determine what suggestions were accorded priority status. We noted considerations and reflections in the groups on the principles used to guide and inform these conversations. While not an exhaustive list, the conversations during the meeting included reflections on equity and justice (who might this help? Might anyone be disadvantaged? What will the impacts be on health inequities?), principles for prioritisation (how many people will this reach? What are the current impacts of the unmet need and potential reach of technologies that might help?), and the wider impacts of technologies outside of health (for example, environmental impact). The need for lived experience input into technological design also emerged as a central concern.

**Fig. 3:**
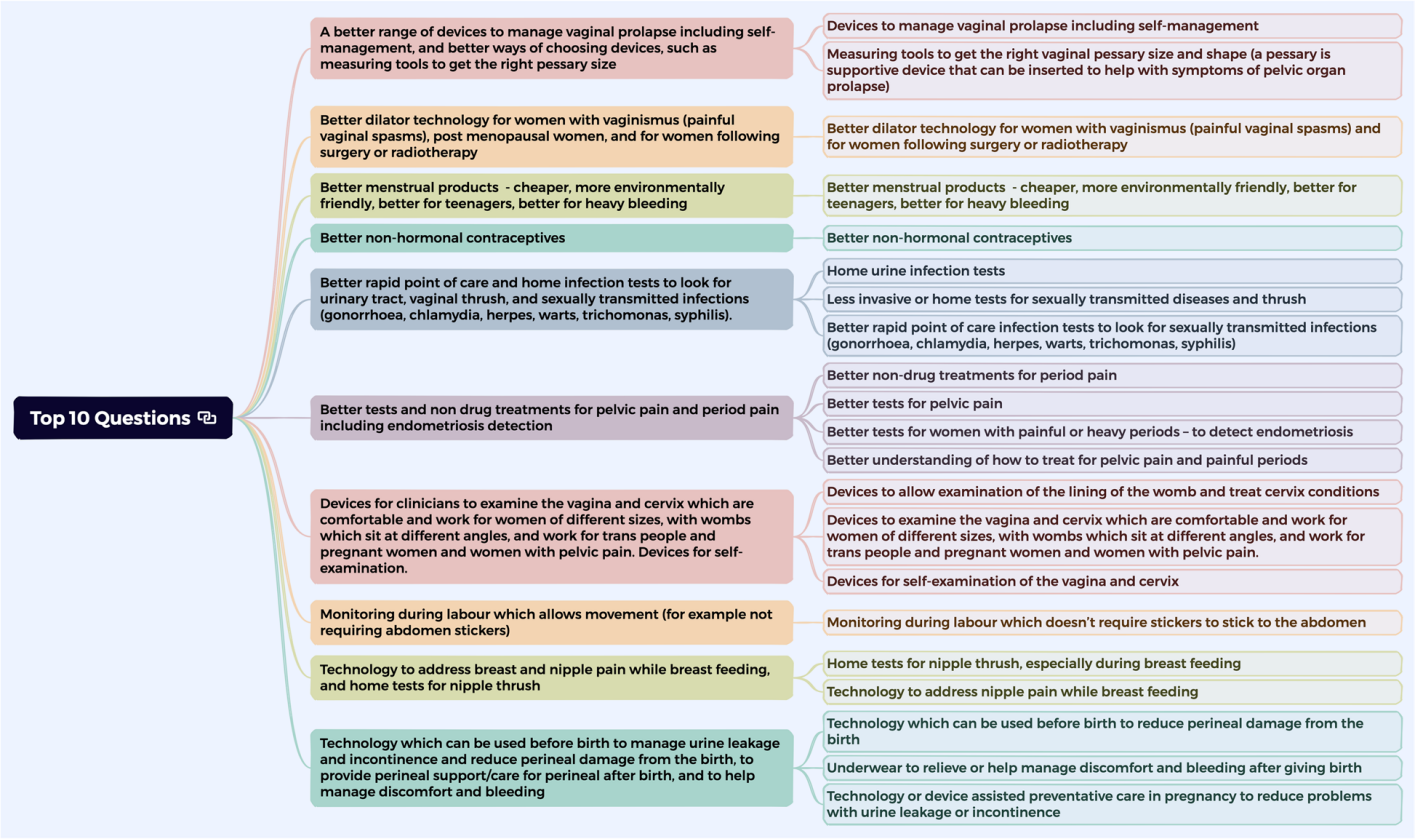
Graphical representation of the top 10 priorities identified, showing the 21 unmet needs that contributed to this top 10

The top 10 agreed priority list, which integrated 21/51 identified unmet needs, is represented in Fig. 3. This includes representing all of the unmet needs that contributed to each of the agreed top 10 priority areas. The top 20 (and the needs that contributed to the top 20) and those that were not taken forward after the first round of ranking are detailed in Appendix F.

### Unmet needs outside the scope of this project

We conducted the priority setting partnership discussions on unmet needs for devices, kit and equipment. However, in the course of the surveys and conversations we undertook, we also heard suggestions about ways in which digital technologies could potentially mitigate against other unmet needs in women’s health and more general concerns relevant to technology development for women. These suggestions are all represented within the initial collated list of suggestions on the project webpage (EMPOWER — NIHR Community Healthcare MIC) and are included in Appendix B.

Some represented unmet needs that were pervasive across all aspects of women’s health. We heard that there is an overarching need to ensure that technologies do not exacerbate or create health inequalities, for example by restricting access to care because of digital poverty, access to equipment or devices, or language, literacy or learning barriers.

Across clinical domains and throughout the life course, access to services and support, which were trauma-informed and accessible, were identified as unmet needs. These suggestions were often contextualised by considerations of how technologies could facilitate service provision and access to care, for example through facilitating appointment booking, enabling information sharing, and supporting shared decision-making. There were suggestions that technology could create platforms to enhance information ownership and sharing, both between patients and healthcare providers and between different care providers. Improving continuity of care by sharing medical records across care providers could enhance safety and patient autonomy and minimise repeated questioning with potential attendant risks of re-traumatisation.

A number of suggestions represented areas of potentially unmet needs which could arise when navigating healthcare encounters. These include unmet needs around helping individuals make sense of symptoms they were experiencing, decision-making to approach or access care, and managing encounters with care providers. Digital tools or apps were often suggested as ways technology could improve care journeys, for example by identifying symptoms warranting medical review, supporting symptom monitoring, collating investigations, or as repositories for advice on treatments and treatment options.

## Discussion

### Summary of findings

Our survey of patients and clinicians elicited 329 suggestions of aspects of women’s healthcare which could be improved by different or improved devices or products. Evidence searches identified three of these suggestions as met needs and we excluded suggestions which were outside the scope of the project. Our priority setting partnership approach with clinicians and patients defined the top ten priorities from the initial long list. Priorities ranged from technologies to improve the management of vaginal prolapse and better non-hormonal contraceptives to better point of care diagnostics for urinary tract infections and sexually transmitted infections. The top priorities suggest far-reaching areas of unmet need across women’s life course.

## Strengths and limitations

### Strengths

We employed a novel and innovative approach to gather public and clinician ideas about both unmet needs and potential technological solutions for women’s health and well-being.

We achieved broad demographic and specialty representation across patient and clinician respondents respectively, with project results made publicly available as a resource to guide future research and development.

By focussing on non-digital technologies, this project adds breadth to the concept of FemTech.

## Limitations

Despite an extensive communications effort and collaborative development process, the reach of our surveys was finite, and the question asked was complex. Factors such as access to internet and English literacy may have influenced the breadth of perspectives captured. We attempted to mitigate against some of the limitations of the written language format of our survey, and the digital literacy that completing it required, by reaching out to community advocacy organisations and offering flexible opportunities for one to one or group conversations.

In addition, our study is limited in its ability to comment on FemTech for trans and non-binary individuals. We did not collect data on gender identity and are therefore unable to assess whether these populations were represented. The language used in the survey content and promotion emphasised women’s health, which may have potentially further limited engagement from gender diverse individuals. Our decision to use the phrasing of ‘women’s health’ was informed by the need for clarity and broad accessibility, with input from public and clinical stakeholders.

## Comparison with existing literature

The James Lind Alliance (JLA) has published principles for priority setting partnerships which seek to bring clinicians, patients and carers together to identify and prioritise unanswered questions or evidence uncertainties that are most important to them, to help guide further research activity and funding [15]. This project was inspired by these principles and methodology, including taking a collaborative and iterative consensus driven partnership process. However, JLA PSPs’ explore research and knowledge uncertainties, whilst our project differed in the type of question we sought to explore.

We wanted to identify what clinicians and the public considered to be unmet or inadequately met needs where technology might help and hear their suggestions for how these needs might be addressed. While there is some overlap between research uncertainties and unmet needs, there are also potential differences, with unmet needs having a broader scope. Therefore, while guided by the principles and steps of the JLA process, we adapted the method for our projects aim. Tailoring the approach to the project is supported by the work of Viergever et al., who offer a thematic checklist for health research priority setting [16]. This includes: considering the context and planning for the project, in which we were supported by our clinician and public project advisers; using a comprehensive approach and considering inclusion, to which end we added discussions and conversations to our survey; determining a shared process towards consensus, in checking the outputs with our participants; and seeking feedback and transparency by publishing our outputs from each step on the project web page and making them freely available.

A systematic review of priority setting in women’s health only identified and included partnership projects using the JLA model [17]. These priority lists tend to be condition or context specific. Therefore, while there are areas of overlap between our project and other PSPs about women’s health, for example: the need to improve diagnostics for endometriosis as a cause of pelvic pain, the focus of our PSP is broader than one condition. PSPs that look specifically at technology have tended to be single condition specific, for example: specific surgical procedures (problematic hip replacement) [18] or interventions/conditions (digital technology for adolescents with inflammatory bowel disease) [19]. These projects identify specific research uncertainties typically, within a single context or condition, and offer detailed needs and suggestions. This project represents a step before that: identifying unmet needs for better technology as suggested by clinicians and the public as priority areas for further detailed exploration.

We have not found any other published work looking at priority setting in identifying which aspects of women’s health needs could be supported by better technologies.

## Recommendations for future research

We have identified a priority list of unmet needs which could be useful to form the basis of future research, to fully characterise the unmet need across a variety of communities in the UK and to develop potential technological solutions that go across the life course. While this project highlights the interest and the need for engagement in this field, there are many more unmet needs and voices to be heard.

These unmet needs represent areas where investment and evaluation are sorely needed. An overarching request from our participants was that technologies should be co-developed and evaluated with those who use them.

## Conclusion

We identified a priority list of unmet needs across a wide range of women’s health and well-being topics, including throughout the life course and across multiple domains of health and well-being. These unmet needs include those associated with female reproductive and sexual health but also extend much wider. They could form the basis of future research and development in women’s health technology.

### Supplementary Information

**Additional file 1.**

**Additional file 2.**

**Additional file 3.**

**Additional file 4.**

**Additional file 5.**

**Additional file 6.**

## Data Availability

We have made our collated findings and materials available through the appendices included within this manuscript.
