# Supplementary material for: What are important areas where better technology would support women’s health? Findings from a priority setting partnership
Source: BMC Womens Health. 2023 Dec 13;23:667. doi: 10.1186/s12905-023-02778-2 (PMC10720144; doi:10.1186/s12905-023-02778-2)
Supplement: Supplementary file 1 — Additional file 1. [file 12905_2023_2778_MOESM1_ESM.docx]

**Appendix A: Surveys used for service-users and healthcare professionals**

**EMPOWER: E**xploring **& M**apping **P**ri**O**rities for **W**omen’s health technology, **E**quipment, kit, devices, and products in health ca**R**e

<https://tinyurl.com/y4dc4erm>

**What will this project do?**

We want to know more about how women’s health and well-being could be improved by having new or better ‘technology’ such as equipment, devices, products, tests or kit women themselves use at home, as well as anything health professionals use to treat or examine women.

We want to know about what might be important or helpful at all of the stages of women’s lives: from being a teenager through to the menopause and beyond. We are interested in all parts of women’s lives, for example in school/education, at work, in relationships and when seeing health professionals for any kind of care or treatment.

Women’s healthcare requires greater focus and funding. It is important that women’s and clinicians’ ideas and priorities are at the heart of technology development.

Women’s voices and views should be central in deciding the most important areas of need. This project, including this survey, will help make that happen.

**Who are we?**

We are a group of health professionals, researchers and women from the University of Oxford.

**How are we going to do this?**

This project will collect women’s ideas about **how** their health and well-being could be improved by better technology. We will also ask the same questions to different types of health professionals who care for women.

Once we get the survey results, we will check whether there is any existing technology which might help with the needs women and healthcare professionals have identified. We will make a resource about the technologies that already exist to support women’s health and well-being, and the evidence and evaluations that under-pin them.

Finally, we will hold a meeting with clinicians and women who have experience with healthcare needs and agree on a priority list of **ten** unmet needs to explore further. We will share this list with partners in industry and work to make and test some of these, ideas so that they can become available to women and the professionals who look after them.

**Data Agreement**

**How will my data be used?**

- By taking part in this survey, you are agreeing for us to use and publish your questions anonymously. **Your information is held and used in compliance with the Data Protection Act 2018**. Any information that you give will only be used in relation to this project.
- The survey is **anonymous**. We will not ask for, and would like you **not** to give us, any information that could identify you. For example, names, addresses, your date of birth, or your phone number.
- We will **not** collect your email address or any information that identifies you from your response to this survey.
- At the end of the survey, there are some additional questions about you – for example, your age, ethnicity and the region of the UK where you live. This is to help us make sure we are getting responses from lots of different people. You **do not have to respond** to these questions if you do not want to.

**Before you start**

Read through this information before agreeing to participate. Tick the ‘yes’ box below if you are happy to continue.

If you have any questions before you start, please contact the researcher using the contact details below.

Your participation is voluntary. If you do decide to take part, you may withdraw at any point before submitting your answers by pressing the ‘Exit’ button or by closing the browser.

This survey takes about **ten minutes**.

**Questions:**

**We want to know about the aspects of women’s health and wellbeing which you think could be improved by different or better technology (equipment, products, resources, kit, devices).**

Your suggestions can include any ideas you think are important for women’s health. They could come from any of your experiences, including your own, the experiences of people you know or care for or anywhere else.

**Please outline up to three things that you think are problems now. For each of these problems, please can you tell us why they are problems for women.**

**If you have ideas about possible solutions for how any of these problems could be improved by better devices, kit, or technology, please tell us about them.**

There are no right or wrong responses, and your suggestions can vary in length and detail.

If you have more than three ideas, you can tell us about the others in the ‘any other comments’ box below.

You don’t need to check or look anything up. We will do that!

**Suggestion 1:**

- *What is the problem?*
- *Why is this a problem?*
- *Do you have any ideas about possible solutions for this problem?*

**Suggestion 2:**

- *What is the problem?*
- *Why is this a problem?*
- *Do you have any ideas about possible solutions for this problem?*

**Suggestion 3:**

- *What is the problem?*
- *Why is this a problem?*
- *Do you have any ideas about possible solutions for this problem?*

***Any other comments:***

**DEMOGRAPHIC INFORMATION**

We will store this information for 12 months before deleting.

1: What is your age?

2: Whereabouts in the UK do you live?

NW

NE

Wales

Scotland

SW

SE

London

Midlands

Northern Ireland

3: What is your ethnicity?

**White**

- *White British*
- *White Irish*
- *White other*
- *Gypsy or Irish Traveller*

**Mixed ethnic background**

- *White and Black Caribbean*
- *White and Black African*
- *White and Asian*
- *Any other Mixed or Multiple ethnic background*

**Asian or Asian British**

- *Indian*
- *Pakistani*
- *Bangladeshi*
- *Chinese*
- *Any other Asian background*

**Black, African, Caribbean or Black British**

- *African*
- *Caribbean*
- *Any other Black, African or Caribbean background*

**Other ethnic group**

- *Arab*
- *Any other ethnic group*
- *Prefer not to say*

**THANK YOU**

Thank you for your contributions to this important topic.

If you would like to help us further by giving us your opinion on which of the suggestions we receive through this survey are priorities, please provide your email address on the web page below:

<https://www.community.healthcare.mic.nihr.ac.uk/research/femtech/view>

Contact information: empower@phc.ox.ac.uk

**EMPOWER: E**xploring **& M**apping **P**ri**O**rities for **W**omen’s health technology, **E**quipment, kit, devices, and products.

<https://tinyurl.com/2p9atv8j>

**What will this project do?**

We want to know more about how women’s health and well-being could be improved by having new or better ‘technology’ such as equipment, devices, products, tests, or kit women themselves use at home as well as anything health professionals use to examine or treat women.

We want to know about what could be helpful at any stage of a women’s life from being a teenager through to the menopause and beyond. We are interested in all parts of women’s lives, for example in school/education, at work, in relationships, and when seeing health professionals for any kind of care or treatment.

Women’s healthcare requires greater focus and funding. It is important that women’s and clinicians’ ideas and priorities are at the heart of technology development.

Women’s voices and views should be central in deciding the most important areas of need. This project, including this survey, will help make that happen.

**Who are we?**

We are a group of women, health professionals and researchers from the University of Oxford.

**How are we going to do this?**

This project will bring together women’s and health professional’s ideas about how women’s health and well-being could be improved by better technology.

Once we get the survey results about the needs women and healthcare professionals identify, we will check whether any technology already exists to help. We will make a resource about the technologies that already support women’s health and well-being, and the evidence and evaluations that under-pin them.

Finally, we will hold a meeting with women and clinicians who have experience with healthcare needs and agree on a priority list of **ten** unmet needs to explore further. We will share this list with partners in industry and work to make and test some of these, so that they can become available to women and the professionals who look after them.

**Who can take part in the survey?**

Any women can take part in this survey.  So can any clinician involved in women’s healthcare, for example GPs, gynaecologists, midwives, health visitors, menopause specialists, obstetricians, physiotherapists, specialist and primary care nurses, and sexual health specialists.

**Data Agreement**

**How will my data be used?**

- By taking part in this survey, you are agreeing for us to use and publish your suggestions anonymously. Your information will be held and used in compliance with the Data Protection Act 2018. Any information that you give will only be used in relation to this project.
- The survey is anonymous. We will not ask for, and please don’t give us, any information that could identify you, such as your name, address, date of birth, or phone number.
- We will not collect your email address or any information that identifies you from your response to this survey.
- At the end of the survey, there are some additional questions about you, your role in women’s healthcare and the UK region where you work. This is to help us make sure we get responses from lots of different people. You do not have to answer these questions if you do not want to.

**Before you start**

Please read through this information before agreeing to participate by ticking the ‘yes’ box below.

You may ask any questions before deciding to take part by contacting the researcher using the contact details below.

Your participation is voluntary. If you do decide to take part, you may withdraw at any point before submitting your answers by pressing the ‘Exit’ button or by closing the browser.

This survey is likely to take **no longer than ten minutes.**

**Questions:**

**We want to know about the aspects of women’s health and wellbeing which you think could be improved by different or better technology (equipment, products, resources, kit, devices).**

Your suggestions can include any ideas you think are important for women’s health. They could come from any of your experiences, including your own, the experiences of friends or people you know or care for, or anywhere else.

**Please outline up to three things that you think are problems now. For each of these, please can you tell us why they are problems for women.**

**If you have ideas about possible solutions for how any of these problems could be improved by better devices, kit, or technology, please tell us about them.**

There are no right or wrong responses, and your suggestions can vary in length and detail.

If you have more than three ideas, you can tell us about the others in the ‘any other comments’ box below.

You don’t need to check or look anything up. We will do that!

Three suggestions: then align and pair (note for George and boxes not for participants).

1: problem 1 why problem 1 and possible solution problem 1

2:

3:

**Any other comments:**

**DEMOGRAPHIC INORMATION**

We will store this information for 12 months before deleting.

1: What’s your role in supporting women’s health and healthcare?

2: Whereabouts in the UK do you work?

Northern Ireland

Wales

Scotland

NW England

NE England

SW England

SE England

Yorkshire

Midlands

London

3: Which best describes the care setting in which you work?

Primary Care

Secondary Care

Community

Multiple settings (please list)

**THANK YOU**

Thank you for your contributions to this important topic.

If you would like to help us further by giving us your opinion on which of the suggestions we receive through this survey are priorities, please provide your email address on the web page below:

<https://www.community.healthcare.mic.nihr.ac.uk/research/femtech/view>

Contact information: [femtech@phc.ox.ac.uk](mailto:femtech@phc.ox.ac.uk)
