# Supplementary material for: What are important areas where better technology would support women’s health? Findings from a priority setting partnership
Source: BMC Womens Health. 2023 Dec 13;23:667. doi: 10.1186/s12905-023-02778-2 (PMC10720144; doi:10.1186/s12905-023-02778-2)
Supplement: Supplementary file 2 — Additional file 2. [file 12905_2023_2778_MOESM2_ESM.docx]

**Appendix B: Collated Summary of Survey Responses**

**Over-arching considerations:**

How can technologies be best utilised and harnessed to enable best care and practice? This could include enabling and educating both patients and HCP.

All technologies used for remote consulting need a trauma informed approach – so that no-one is put at risk or made more vulnerable.

All technologies need to be evaluated with a health inequalities lens – we need to ensure we do not exacerbate or aggravate inequalities by worsening access to care because of digital poverty, lack of data, device limitations, or language, literacy or learning barriers.

Online resources exist and there are many of them – but how to integrate them so that they can be meaningfully used to improve knowledge and care is less clear. Can they be curated and presented differently?

How, when, where and for who will digital approaches help enable care, self-efficacy, and evidence based practice? For which conditions? On what platforms? With which safety and privacy provisions?

Considering the needs of carers and how these can be supported aligns with all aspects of researching and understanding women’s health.

Continuity of care is hugely valuable, but being systematically eroded. Technologies need to not damage this further, or be offered as solutions that distract from a focus on this. However, alongside relational continuity, information continuity may help support care and this can be explored, including to minimise repeated questioning and re-traumatisation.

**Service level considerations:**

There is a need for research and resources for all aspects of health where gender or sex may influence presentation, prevalence, treatment or care. Examples given include auto immune disease, osteoporosis, thyroid disease, type 2 diabetes (for example aligned with PCOS) migraines, benign intracranial hypertension, and heart disease.

Research and resources that can include and be specific for women’s health and wellbeing are needed – including mental health, exercise, diet and weight management.

Ways to map symptoms, including across systems, and involve women in advocating for their care are important. Home diagnostics and symptoms diaries and patient held records may help. NICE guidance, centralised resources, better education at school and medical school could help.

Would enabling all primary care clinicians to respond to women’s health needs help? Or might developing champions or women’s health community hubs better meet women’s needs? How might this be achieved? What community and specialist services need to evolve or be developed to support this? What are their educational and resource needs? How might technologies help bridge or moderate these functions, in ways that enable women and HCP (without overwhelming them).

What is the role of continuity of care in women’s health? How could this be optimised? Could technology help? Primary care holds women’s health through the life course and is central to it. What could help them do this? And how and what technologies could help? Would prompts help?

**Menstrual bleeding and cycles:**

Information and education needs to include what is (or isn’t) normal, which resources and apps are reliable, tools for those with irregular periods, and pre-menstrual symptoms. Knowing when symptoms are part of normal menstruation and when they might suggest endometriosis is difficult. Any tools that could help support this are needed. This could include information and awareness, apps for monitoring symptoms or to support conversations with clinicians, early diagnostic tests (bio-markers or imaging). Could technology and apps be used to enable women to find out about and participate in research?

There is a need to know more about the support for period bleeding and symptoms towards the end of menstruation and into the peri-menopause. Could this be helped if women could monitor urinary hormone levels or other markers? Could this help guide evidence based care? What about other approaches such as smart watch or bracelet temperature monitoring?

Non-bleeding aspects of menstruation are also important – for example acne. How does this interface with hormonal fluctuations and could measuring these help? There are also health concerns correlated to different points in the menstrual cycle, such as migraines.

Could home hormone measurement or monitoring help with managing menstrual pain, menstrual health, irregular periods, polycystic ovary syndrome (PCOS), fertility, menopause, puberty.

There is a need for more knowledge about how to treat menstrual pain, whether it is or is not endometriosis related. This includes evidence about whether hormonal treatment helps or masks symptoms. More information about the risks and benefits of all treatments would help. Non-hormonal and non-drug options such as TENS (transcutaneous electrical nerve stimulation) machines are important to consider.

PCOS is a comparable area where there is a need for better information and diagnostic guidance. There is also a need for more research into fibroids. Miscarriage needs greater research. Could home monitoring tests be of help? What support and resources would help?

Menstrual products need to be developed to mitigate against period poverty and environmental impacts. This needs to include packaging and products. Apps to tell you what is cheapest or free and where to get them could help. Apps to help change products often could also make menstrual products safer.

There needs to be better equipment for dignified and safe disposal of menstrual products. School toilets need to be better designed and equipped. Menstrual products could be designed for a wider range of needs – heavy menstrual bleeding, teenagers learning to use products, those that find them hard to use. There is a need for better products for heavy menstrual bleeding. These need to be comfortable, environmentally friendly and reliable. Could these be adapted to measure flow?

**Pain:**

Pain in women is undertreated and underdiagnosed, despite greater prevalence. What would help this journey? Communication, training for HCP, resources, bespoke analgesics, better dosing regimens, innovative modes of delivery (Tampax, intra-vaginal delivery method)

Pelvic pain is debilitating and services are not uniformly available, which is wrong and un-just. This can include women experiencing marked pain with sex for many years and these women can be left without access to specialist support for this. This includes better recognition and care for vulvodynia.

Pelvic pain care requires a better range of options or a fuller treatment pack for the NHS that includes better dilators, with a vibrator and kit for relaxation and stretching would help. Women need better dilators for pelvic floor pain associated with surgical or radiotherapy treatment to the vulva. They also need specialist devices to help with vulval pain or vaginismus.

Physiotherapy and pelvic floor devices to help with vaginismus and pelvic floor pain are needed.

Pelvic inflammatory disease is usually associated with a sexually transmitted infection. This can be given as a provisional diagnosis of exclusion, often in surgical or A&E units, where a woman has (as yet) undiagnosed menstrual or pelvic pain. If the ultrasound and pregnancy tests are point of care and normal (negative), then swabs are taken but no results are available, women leave hospital with a potential diagnosis. This then generates work in communicating the results, by which time, there may have already been adverse consequences for her relationship and well-being. Point of care tests for common STIs from swabs would significantly improve this.

**Screening and health promotion:**

Smears could be undertaken at home, with self-taken HPV swabs. Who would this benefit? What might be the unintended risks? Who might it not benefit?

At present, with in person cervical screening, even if you want to attend for it, it is difficult to book an appointment at the right time in your cycle and at a time that is convenient. Could this be centralised (like the vaccine booking) to make it easier for women to find appointments they can attend? Designated slots, online booking, or accessible well women clinics, supported by adequate information could help.

Mammography is painful and disagreeable, and the exposure to x-rays carries risk in their own right. Alternative screening modalities with less risk, and more comfortable methods of screening would be beneficial for women. This could be as simple as developing a softer cushion rather than a plate to compress the breasts when the image is taken. Could women become involved in the design process?

Knowing what is normal, and a better understanding of the range of normal would help women understand when to go to the doctor. Better online resources about what the whole range of normal breasts and vulvas look like would help women become empowered to know when to seek advice and why. These could link or be part of a women’s health app that would tell women what to monitor and when to seek advice across a range of women’s health and gynaecological issues. This could be linked to a woman’s life course, with alerts and reminders relevant to her age or stage of life.

Educational materials on vaginal anatomy such as 3D scanning of one’s own genitals could be a constructive way to teach anatomy and sex education. This could be especially useful to women who have given birth and are concerned about perineal damage.

**Pelvic floor care and prolapse:**

Pelvic floor problems are common and can be embarrassing and debilitating. This includes stress incontinence and leaking with exercise. Well woman checks that ask about prolapse – and don’t normalise it!

The NHS App Squeezy helps and could be promoted for pregnant and post-natal women – but it would be good to have well evaluated technologies to support continence and pelvic floor care.

An app to tell people where there are public toilets and access to toilets could help people feel confident to go out.

Better devices to manage vaginal prolapse – including devices women can use themselves at home. Biofeedback devices for pelvic floor exercises can be difficult or boring to use. Would it be possible to make a device that is smaller and easier to insert and remove?

It is hard to know accurately what size pessary to trial or order for a woman based only on examination. A better measuring tool or guide would be helpful. This could include information about other variables to consider. This might make fitting more tolerable and more effective.

Better assessment would help plan treatment – and may help with shared understanding of what the problem is and how exercises can help. Point of care perineal ultrasound may help with this, including giving biofeedback.

Overactive pelvic floors often go undetected, and may be made worse by standard pelvic floor exercises. A device could help with relaxing the pelvic floor and minimising urinary incontinence.

**Urinary symptoms and incontinence:**

There are inadequate services to support women with incontinence and this adds to the difficulty of managing these difficult symptoms. Women need better access to care and products. Reducing the stigma of incontinence would also help women.

Pregnancy and the post-natal period are important times when urine leakage develops or becomes problematic and we need to know more about opportunities for pelvic health improvement.

One difficulty that can compound the investigation of urine leakage, is that women may be unsure whether they have dampness because of leakage or vaginal discharge. A pad that changed colour in the presence of urine would help – and could also help monitor the impact of interventions to help pelvic floor health and incontinence.

**Infections:**

Could home diagnostics for UTI (urinary tract infection) help? How would this work for acute UTI? What about recurrent or chronic UTI?

POC or home taken tests for thrush would help guide treatment. This includes vaginal and nipple thrush.

Could there be better ways to manage and diagnose vaginal discharge? Pants, panti-liners?

POC tests for pelvic inflammatory disease and access to rapid point of care testing and less invasive testing for STIs would help – for men and women. Examples include chlamydia and trichomonas

A habit tracking diary could help with recurrent BV and thrust infections by identifying a link to lifestyle activities.

It can be difficult to keep ointments in place on the vulva. Could a material like silly putty help with keeping ointments or corrosive treatments in the correct area?

**Contraception:**

A better range of options for women are needed. This could include non-hormonal means (which could include male burden contraception) and hormonal methods (better range of low dose pills, different formulations for POP methods, different designs vaginal, better colour range for patches).

Technologies could enable and support women in their choice and use of contraception. Could hormone levels help individualise care? Including navigating side effects including bleeding and mood. This could include the possible impacts on menstruation from different medications or conditions or personal variables such as BMI.

Could technology support access to care (for example a platform to upload information) and promote safe care (alerts to expiry etc).

IUD (copper coil) insertion can be difficult and painful. This leads to negative messaging and puts people off, though it is a very safe and effective method. Less painful modes of insertion would help. Copper coils are not well packaged for insertion. There is currently huge variation between IUD devices, which can be confusing. Could these be improved? For example, could it be made more like the hormonal coil (IUS) – or could there be a more universal insertion device – the multiple devices make it difficult for fitters, especially new fitters. Can the devices be thinner or better designed for nulliparous women? Could tenaculums be re-designed and re-developed? Could pain relief be self-inserted by women prior to their appointments?

Checking for the thread on a copper or hormonal coil can be uncomfortable for some women, which can make it difficult to know if the device is in place. Could design changes like placing a bead at the end be made to make the cut end easier to identify?

The experience of IUD insertion could potentially be improved through technologies like TENS machines or virtual reality headsets. This could potentially be used in a way that gives women more control over their environment. Tools like a colour changing chart to communicate how pain levels vary at different points in the procedure.

**Fertility:**

Technologies to help people have babies that are cheaper and become more available could help.

Technologies to promote women’s autonomy within healthcare would be beneficial. This could include an app to hold all of women’s knowledge and information. This could include home hormone monitoring.

Less wasteful plastic and single use kit in ovulation and pregnancy tests and equipment is important.

How could we optimise non-invasive or home ovulation testing. There are bracelets that monitor body temperature – could smart watches do this? How reliable are they?

**Pregnancy:**

Pain medications and interventions in pregnancy that are effective but that don’t impact negatively on the mother’s well-being and autonomy would be beneficial.

Any interventions or technologies that address maternal mortality and morbidity are desperately needed. It would be important to ensure that these are universally evaluated and effective, and could then help address known inequalities in outcome experienced across setting and ethnicity. These should be able to be made available across the world and in settings where they are most needed. Could we use technology to help women become more empowered and enabled in their pregnancies? For example by using them to do personalised risk calculations, and then given advice and reminders, for example about folic acid, medications, immunisations in pregnancy and exercise.

There is a need for more evidence and resources about the impacts and safety of medications in pregnancy, to support maternal choice and well-being.

Technology used for monitoring that could enhance the safety and wellbeing of pregnancies would be welcomed and is much needed. This could include technologies that give alerts when there are opportunities for intervention or help is needed. Possibilities are devices that women can use at home to record movements or heart rate of the baby – but these need to be fully assessed and evaluated for a range of women and pregnancies including consideration of unintended consequences and potential harms (including not seeking or accessing care, recording maternal and not fetal heart rates, limitations of BMI and multiple pregnancies).

A cheap and accessible way of knowing whether you are leaking amniotic fluid (or urine) in later pregnancy would be very helpful. One product is a panti-liner that changes colour but these are very expensive and not easily available in the UK.

Having universal NHS maternity notes that transition between care settings (different NHS trusts and also primary care) would enhance care and safety – and it would be easier to not have to carry a folder. What are the online solutions and suggestions?

How could technology help optimise antenatal care? For example advising about healthy diets, exercise, and folic acid.

Testing needs to be legislated and evaluated, including for unintended or unanticipated harms. Examples where this could be important include very early pregnancy tests (biochemical pregnancy then miscarriage before a significantly missed period associated with unnecessary worry and concern – do we know the evidence for the impact and significance of these?) or 4d USS scanning in pregnancy.

Women with recurrent miscarriage are advised to start progesterone as soon as they are pregnant – so access to accurate and reliable early tests are is important. In this context, how early can tests become positive and how could this link to access to medication or advice to start it? Could POC home tests help quantify progress of pregnancy through b-HCG levels? This could help women monitor themselves and be enabled to seek help and advice but also could help emotionally prepare for transition. Late diagnosis of missed miscarriage can be devastating. Could tech help reduce this time period? Could early routine scans help? Access to earlier scanning or testing?

There is a need for supportive resources and guidance to enhance conversations about early pregnancy and about pregnancy loss. This includes when communicating about access to care.

Home BP monitoring in pregnancy should be offered – at present this is variably available during pregnancy, but then there is no monitoring or devices available post-natally. There is also not enough known about the risk of long-term cardiovascular and hypertension outcomes. Should all pregnant women be given a BP monitor to use in pregnancy and beyond?

CTG monitoring in pregnancy can be complicated by transducers not sticking.

Methods and devices to promote perineal care and reduce the risk of trauma need to be evaluated – including effectiveness, for whom they do (and do not) work and risks, including any unintended consequences. This includes Epi-no devices, Ani-ball and perineal massage guides.

**Post-natal care:**

Post-natal mental health is hugely important. Investing in support, including children’s centres and facilitated social and support groups would help. Mentorship and buddying might help. Could technologies help connect people, offer support, or follow them up?

There is a need for wider and more accessible support for post-natal pelvic floor health. This needs to be promoted and offered flexibly so that it can be available for all women. This could include a range of formats, including online, in person, and in different languages. Apps could be part of this, but devices that women can use to maintain and develop pelvic floor health need to be evaluated and made widely available. A device that women can use at home to measure their pelvic floor strength and then monitor their own physiotherapy for the post-natal period would be valuable.

Kangaroo shirts that help new mothers bond with and care for their babies – and also help with abdominal and pelvic pain post-delivery. These might also help mitigate against some of the challenges of staffing on NHS maternity wards.

Better underwear for post-natal comfort and bleeding – akin to period pants but designed for this.

An application could help women track and gain information about lactation amenorrhea.

There are often gaps in post-natal care. This can include lost opportunities for follow up for conditions diagnosed in pregnancy (for example gestational diabetes). This can also include mental health follow up, and follow up after obstetric trauma. Could technologies enable women to be active in their follow up encounters? And be used to facilitate communication between primary and secondary care? And between obstetrics and midwifery? And midwifery and health visiting. This also includes screening for hypertension after a diagnosis of pre-eclampsia.

Devices promoted for new families to promote feeding, infant care, and infant sleep need to all be fully evaluated. This needs better regulation. New mothers are vulnerable to marketing and the post-natal period can be a highly emotionally charged time. Products that rock or swaddle or carry infants may affect expectations and understanding about normal crying and sleep expectations and be associated with harms. These need to be better evaluated, documented and communicated about.

Many women could benefit from postnatal rehab like core strengthening to prevent future issues with back pain and pelvic floor dysfunction. Abdominal issues like diastasis recti also require more attention. Postnatal services should be better funded and women should be signposted to more online resources.

**Breastfeeding:**

Treating nipple thrush is important and difficult. It is hard to let the gel air dry on nipples – and if they are in contact with leaked milk, then the treatment does not remain in place. Finding ways to optimise treatment and also minimise contact with warm leaked milk which aggravates the difficulties would be very helpful – for example a shield that could hold the treatment gel in place and ideally also collect drip milk. If it was possible for this to be washable and re-usable, that would be better for the environment.

Knowing how to advise and support mothers who are not able to breast feed, or to breast feed fully with flexible and supportive messaging that meets the needs of all – including those who would want to breast feed, but are not able to.

Shields for nipple pain or to collect drip milk could be better designed so that they work and do not leak.

There are many devices for extracting or pumping breast milk. Which ones to use when/for which women is not known. What works for mastitis? What could help them work well?

**Intimate examination and speculums:**

Speculum examination can be painful and uncomfortable, and can be seen as hostile and off putting. Women have little agency. Could there be better designed speculums for self-examination or greater choice and range of size, shape, and coloured speculums that are less intimidating and uncomfortable for women to experience? Could they be better designed for tilted or retroverted uteruses. Multiple attempts and difficulties can make this very uncomfortable and off putting. Could better speculum design help? This could include speculum design for women of differing sizes and builds. Can we involve women in designing better speculums? Could they be less cold?

Part of better speculum design would include self-examination options and trauma informed design and care, for women who have pain and trauma associated with intimate examination. These could be co-developed by women with these experiences.

Speculums that meet the needs of non-binary and trans individuals are desperately needed.

Surgical gowns are not designed for women, and this compounds distress and indignity when being examined or going through medical procedures. Gowns which are designed for women would help.

Could hysteroscopies (camera imaging of the cervix and uterus) be conducted without the use of a speculum?

Could a device allow patients to apply their own vaginal analgesia (pain relief) prior to a coil fitting? The current method of applying pain relief is difficult to use on those with obese or tall bodies. Could this be addressed in an improved design?

**Menopause:**

Could there be a better way to predict or diagnose the menopause for individual women, than variable hormone levels and waiting until it has already completed? This could support earlier considerations and conversations and access to supportive care and treatment. Could home hormone testing and better understanding of hormone fluctuations help?

Better research and information for women before and during the menopause, curated and updated in an accredited website or resource is needed. The information that is there is quite ‘generic’ and it is hard to find personalised reliable or in depth information. This could include both hormonal treatments (HRT and testosterone) and impacts of surgical procedures. This could also include the wide range of potentially associated symptoms and experiences and other conditions (for example fatigue, depression, acne, arthritis) that might change or become more prominent at the time of the menopause, and how these can be managed effectively and in an evidence based way.

Equity in research and resources for libido and sexual wellbeing during the menopause. This includes vaginal dryness (and devices to apply treatments). For libido, Viagra is available over the counter and on the NHS for men, yet there is little research about testosterone for women and it can be very difficult to get hold of. This means that some go privately which creates injustice.

The impacts of the menopause on mental health and mental well-being are not adequately understood or supported. Knowing how this interfaces with mental health is under-researched, with concerns that this contributes to long periods of sub-optimally managed symptoms and difficulties. Could access to hormone checking and monitoring and better diagnostic understanding help?

A platform that allows women to track and share their symptoms and then share these with GPs – and perhaps link to resources for both could be valuable. Perhaps something akin to the Zoe app would increase knowledge and understanding about the menopause?

Better tests to predict, prevent or diagnose osteoporosis in women would be important to develop.

Post-menopausal vulvovaginal atrophy causes significant pain, including affecting sex and urinary function. Topical oestrogens help, but the current modalities of delivery are problematic, either because they are very greasy, making them difficult to use, or need a lot of single use plastic. Treatment would be more accessible if vaginal oestrogens were available over the counter. Talking about vulvovaginal atrophy can be difficult, and there is a lack of information and awareness.

**Other considerations:**

NHS wide systems for people to enter information about outcomes and provide feedback including about complex care journeys that could be used to provide both specific feedback to individuals involved in those care journeys and also case based education.

The rigidity of gender in NHS IT systems impacts on access to screening and care for non-binary and trans people and there need to be changes made to address this, including in the language used in communications and by professionals.

We need better individualised tools that measure people and lead to informed conversations about health and weight. BMI was not developed for or evaluated in health settings or in women (or people of colour). Conversations about weight can act as barriers for people accessing care. This includes excluding many individuals who would want and would benefit from care for weight management and eating disorders. There is a disconnect between primary care and services for weight and eating difficulties is problematic. Women’s bodies are different – could exercise and diet regimens be developed and tested for women which could then be individualised? What do we know about the impact of hormonal cyclicity on weight and weight management? Could hormone profiles inform this or allow women to have more individualised care?

Making women safer in all contexts and situations is important. How could technology help enable women to be (and feel) safe on the streets? How could education, apps, or other help?

Research happens in silos – could technology help bring things together in a more accessible and usable way?

Clothing design – including sports clothing design – needs to be developed flexibly to meet the needs of a wider range of women’s builds. Clothing made akin to men’s systems (waist, length) would help. Wetsuits designed for women with breasts and hips would be appreciated.

Object and furniture design needs to be for women also – please can set up of things like ironing boards take women’s height and arm span into account? And look again at kitchen counter height and cupboard options. And can’t there be women’s adaptions or options for seat belts. Why is the default one that is designed for men? It can be hard to reach car pedals. Can seat position be reviewed? Or floor inserts that are robust and safe to use for access to pedals? Mobile phones could be designed for women’s hands and women to hold and use. Can baby scales be made to better accommodate smaller babies?

Medications should be trialled in women, including dose adjustments for body size and consideration of interactions with hormones.
