# Supplementary material for: What are important areas where better technology would support women’s health? Findings from a priority setting partnership
Source: BMC Womens Health. 2023 Dec 13;23:667. doi: 10.1186/s12905-023-02778-2 (PMC10720144; doi:10.1186/s12905-023-02778-2)
Supplement: Supplementary file 3 — Additional file 3. [file 12905_2023_2778_MOESM3_ESM.pdf]

Appendix C: Tool used to rank service-user and clinician survey responses

EMPOWER ranking

These are the suggestions for areas where new or better technologies could support women's health and well-being that we have heard during the EMPOWER project so far.

We would love your help in ranking these suggestions, to help us work out which ones should be priorities for further work.

Please arrange the suggestions placing the ones you think are most important at the top of the list, by dragging and dropping them into your priority order. You can focus on your top three if that is easier. The suggestions are in groups – you are welcome to arrange as many or few lists as interest you.

Please note that we have not included technologies such as Apps in this list, however they are of huge interest to us, and will be evaluated and considered. These suggestions about the need for better knowledge, resources, and information are included in the full response list detailing what we heard, which you can access here:

https://www.community.healthcare.mic.nihr.ac.uk/files/reports-and-resources/what-we-have-heard.pdf

If you would like to add anything, please complete our survey before 10 May 2022:

https://www.community.healthcare.mic.nihr.ac.uk/research/femtech
Please contact us on empower@phc.ox.ac.uk if you have any other comments or feedback.

You are welcome to skip sections you do not want to comment on by clicking 'next' and moving to the next section.

Menstrual bleeding and periods

1. Menstrual bleeding and periods

Please drag and drop these statements from most important at the top, to least important at the bottom.

You can focus on just your 'top three' if it's easier.

- Better tests for women with painful or heavy periods – to detect endometriosis
- Better tests for women with painful or heavy periods – to detect fibroids
- Better tests for women with irregular periods – to diagnose polycystic ovarian syndrome
- Better non-drug treatments for period pain and pelvic pain
- Cheaper menstrual products
- More environmentally friendly menstrual products
- Menstrual products for heavy menstrual bleeding
- Menstrual products which measure flow
- Better range of options for menstrual products for teenagers starting their periods.

Pain in women

2. Pain in women

Please drag and drop these statements from most important at the top, to least important at the bottom.

You can focus on just your 'top three' if it's easier.

- Device to measure hormones as a trigger for migraine
- Better tests for pelvic pain
- Better dilator technology for women with vaginismus (painful vaginal spasms) and for women following surgery or radiotherapy
- Better ways to administer pain relief for pelvic pain

Screening and health promotion

3. Screening and health promotion

Please drag and drop these statements from most important at the top, to least important at the bottom.

You can focus on just your 'top three' if it's easier.

- Technology to allow women to take their own swabs and smears
- More comfortable breast screening tests
- Breast screening tests which don’t involve X-Rays
- Ways to visualise your genital anatomy and pelvic floor to help with education and pelvic floor exercises
- Diagnostics for osteoporosis

Pelvic floor care and prolapse

4. Pelvic floor care and prolapse

Please drag and drop these statements from most important at the top, to least important at the bottom.

You can focus on just your 'top three' if it's easier.

- Technologies to support pelvic floor care – smaller, easier to use than current models, allowing biofeedback
- Devices to manage vaginal prolapse including self-management
- Measuring tools to get the right vaginal pessary size and shape (a pessary is supportive device that can be inserted to help with symptoms of pelvic organ prolapse)
- Technology to assess pelvic floor disorders
- Pelvic floor relaxing devices
- Applicators for topical oestrogen

Urinary symptoms and incontinence

5. Urinary symptoms and incontinence

Please drag and drop these statements from most important at the top, to least important at the bottom.

- Tests which can show whether fluid is urine or vaginal discharge to help investigating incontinence
- Better pads and products for urine leakage
- Technology or device assisted preventative care in pregnancy to reduce problems with urine leakage or incontinence

Infections

6. Infections

Please drag and drop these statements from most important at the top, to least important at the bottom.

You can focus on just your 'top three' if it's easier.

- Home urine infection tests
- Home tests for vaginal thrush
- Point of care tests for STIs – for both men and women. Examples include chlamydia and trichomonas
- Better rapid infection tests to look for STIs in the context of possible pelvic inflammatory disease.
- Less invasive or home tests for sexually transmitted diseases
- Something to help keep ointments in the right place on the vulva or nipple (and stop them going onto skin)
- Home tests for nipple thrush, especially during breast feeding

Contraception

7. Contraception

Please drag and drop these statements from most important at the top, to least important at the bottom.

You can focus on just your 'top three' if it's easier.

- Better non-hormonal contraceptives
- Better designs for hormonal contraceptives
- Hormone tests to help women use contraception tailored to their needs and avoiding side effects
- Better technology to reduce the discomfort of inserting a coil or Mirena coil
- Pain relief that could be self-inserted prior to coil fittings
- A coil that would allow women to easily check if it is in the right place
- Male contraceptive pill – the burden falls solely on women
- Varied size contraceptive ring
- Contraceptive patches in a variety of skin tones
- New contraception forms such as reversible non-surgical sterilisation

Fertility

8. Fertility

Please drag and drop these statements from most important at the top, to least important at the bottom.

- Tests to help people who struggle to conceive know when they are most fertile
- More environmentally friendly pregnancy and ovulation tests

Pregnancy

9. Pregnancy

Please drag and drop these statements from most important at the top, to least important at the bottom.

You can focus on just your 'top three' if it's easier.

- Home monitoring devices for pregnancy to alert mum if something is wrong and she needs to be assessed
- Tests to confirm whether there is a leak of amniotic fluid (waters) in pregnancy
- Home tests to help diagnose and monitor early pregnancies in women who have had repeated miscarriages previously
- Monitoring during labour which doesn’t require stickers to stick to the abdomen
- Technology which can be used before birth to reduce perineal damage from the birth
- Underwear to relieve or help manage discomfort and bleeding after giving birth
- Blood pressure monitoring to mitigate the risk of pre-eclampsia
- Home Doppler ultrasound devices for fetal heartbeat monitoring
- Device for self-insertion of dinoprostone (medication used to induce labour)
- Technology to support recovery from diastasis recti (separation of the abdominal muscles during pregnancy)

Breastfeeding

10. Breastfeeding

Please drag and drop these statements from most important at the top, to least important at the bottom.

You can focus on just your 'top three' if it's easier.

- Technology to make nipple thrush cream easier to apply and keep in the right place
- Technology to address nipple pain while breast feeding
- Better technology to collect dripping breast milk

Intimate examination and speculums

11. Intimate examination and speculums

Please drag and drop these statements from most important at the top, to least important at the bottom.

You can focus on just your 'top three' if it's easier.

- Devices to examine the vagina and cervix which are comfortable and work for women of different sizes, with wombs which sit at different angles
- Devices to examine the vagina and cervix which are comfortable and work for trans men and pregnant women and women with pelvic pain.
- Devices for self-examination of the vagina and cervix
- Surgical gowns designed for women
- A device to examine the womb and cervix without needing a speculum
- Devices to let women put local anaesthetic or pain relief into their cervix or vagina before procedures – including speculum, coil fitting or examinations of the womb.

Menopause

12. Menopause

Please drag and drop these statements from most important at the top, to least important at the bottom.

- Tests to identify menopause better and earlier
- Better tests to predict, prevent or diagnose osteoporosis in women
- Better ways to deliver hormones into the vagina to treat dryness and pain due to the menopause
- Device to monitor hormones as a cause of symptoms of menopause
- HRT patches which don’t fall off
- Varied colour HRT patches (for all skin colours)

Other considerations

13. Other considerations

Please drag and drop these statements from most important at the top, to least important at the bottom.

You can focus on just your 'top three' if it's easier.

- Better ways to measure women’s weight and advise them about diet
- Better ways to diagnose and manage conditions that affect women more often than men
- Better ways to diagnose and manage conditions that affect women differently from men
- Technologies that help keep women safe when they are out
- Sporting equipment that is designed for women, and for a range of women’s shapes and sizes

This content is neither created nor endorsed by Microsoft. The data you submit will be sent to the form owner.

Microsoft Forms
