# Supplementary material for: What are important areas where better technology would support women’s health? Findings from a priority setting partnership
Source: BMC Womens Health. 2023 Dec 13;23:667. doi: 10.1186/s12905-023-02778-2 (PMC10720144; doi:10.1186/s12905-023-02778-2)
Supplement: Supplementary file 4 — Additional file 4. [file 12905_2023_2778_MOESM4_ESM.docx]

**Method:**

1.       A google search for the product / tech. (Scoping)

a.       Use a private browser so your search history doesn’t affect results

b.       You could do an advanced search – limiting results with specific terms for example.

2.       Evidence search that the product / tech resolves the problem. (Literature search)

a.       E.g. PUBMED search using MeSH and title/abstract search terms.

b.       Build search strategy using terms for product, terms for the condition or population, and terms for the problem.

3.       Search for products in development which might solve the problem.

a.      Science Citation Index via Web of Science (https://www.webofscience.com/wos/woscc/basic-search)

b.       [arXiv](https://arxiv.org/) - computing science

c.       [Europe PMC](https://europepmc.org/) - limit search to preprints from a range of archives

d.       [medRxiv](https://www.medrxiv.org/) - health and medicine

4.       If the evidence search (2) suggests the product doesn’t help, search for products in development (3) which might address the shortcomings.

The technology readiness score system is a version of the method designed by NASA, which has since been adapted to use for medical devices. Need level was evaluated based on the technology readiness score, with scores of 8 or 9 indicating a need was met. This is summarised in supplementary table 1.

Appendix D, Supplementary Table 1

| Readiness level | Description |
| --- | --- |
| 1 | Idea formulation and initial research |
| 2 | Applied research performed |
| 3 | Project plan and schedule devices |
| 4 | Design stage |
| 5 | Proof of concept / design refinement |
| 6 | Preclinical evaluation |
| 7 | Clinical trials and technology transfer |
| 8 | Clinical trials completed |
| 9 | Distribution and Marketing |

*Supplementary Table 1: technology readiness scoring system*

The classifications of ‘unmet need’, ‘partially met need’, and ‘met need’ were designated by the research team based on the evidence of technology availability and appraisal. This is summarised in supplementary table 2.

Appendix D, Supplementary Table 2

| Need level | Description |
| --- | --- |
| Unmet need | Little evidence of products to meet the need, or low readiness |
| Partially met need | Some evidence that products to meet the need, intermediate level of readiness of a product which meets the need |
| Met need | Strong evidence that a device or technology with a high level of readiness meets the need |

*Supplementary Table 2: Summary of matching of need category to technology availability and appraisal*

Citation for technology readiness score:

1. Technology readiness level definitions - NASA [Internet]. NASA; [cited 2023 May 1]. Available from: https://www.nasa.gov/pdf/458490main_TRL_Definitions.pdf

2. Understanding technology readiness level for medical devices development [Internet]. Tempo; 2021 [cited 2023 May 20]. Available from: https://www.tempoautomation.com/blog/understanding-technology-readiness-level-for-medical-devices-development/
