# Supplementary material for: What are important areas where better technology would support women’s health? Findings from a priority setting partnership
Source: BMC Womens Health. 2023 Dec 13;23:667. doi: 10.1186/s12905-023-02778-2 (PMC10720144; doi:10.1186/s12905-023-02778-2)
Supplement: Supplementary file 5 — Additional file 5. [file 12905_2023_2778_MOESM5_ESM.docx]

| **Medical Technology Type** | **Problem(s) to be Addressed** | **Search Terms Used** | **Trials Identified** | **Systematic Reviews Identified** | **Technology Readiness Level** | **Need Level** | **Notes** |
| --- | --- | --- | --- | --- | --- | --- | --- |
| Non-compressive breast cancer imaging | Pain from compression of the breast tissue between two plates during mammography | 'breast cancer', 'invasive ductal carcinoma' AND 'compression', 'imaging', 'scanning', 'screening', 'mammography' AND ‘comfort’, ‘compression’, ‘non-compressive’ | Koning Cone Beam CT device with USA FDA approval, clinical trials ([1](https://clinicaltrials.gov/ct2/show/record/NCT01952717?term=Koning+3D+Breast+CT&draw=2&rank=1),[2](https://clinicaltrials.gov/ct2/show/results/NCT01880580?term=Koning+3D+Breast+CT&draw=2&rank=2),[3](https://clinicaltrials.gov/ct2/show/NCT03861221?term=Koning+3D+Breast+CT&draw=2&rank=3)) have been conducted but findings only reported for [pilot study](https://pubmed.ncbi.nlm.nih.gov/20651210/) which also found [improved patient comfort](https://pubmed.ncbi.nlm.nih.gov/22439131/) | None | 8 | Met | Koning CT device currently available outside of the UK. Trials were commercially funded and have limited reporting, but have yielded FDA approval. Need for further research and systematic reviewing.  Alternative technologies existing such as breast MRI and ultrasonography, though these are typically adjunct to mammography. |
| Self-test swabs for cervical smears | Unpleasant to undergo standard smear, discomfort and pain, access and scheduling issues creating barriers to care | 'cervical cancer', 'cervical squamous cell carcinoma', 'cervical adenocarcinoma' AND 'Self-swab', 'self-sampling', 'home swab', 'home smear testing', 'self-test', 'mail-in test' | NHS-based [feasibility study](https://pubmed.ncbi.nlm.nih.gov/27235844/) and randomised controlled trials ([1](https://pubmed.ncbi.nlm.nih.gov/21343937/),[2](https://pubmed.ncbi.nlm.nih.gov/25403717/)) of self-swab cervical screening | [International systematic review of cervical self-sampling](https://www.frontiersin.org/articles/10.3389/fpubh.2018.00077/full) | 9 | Met | Successful trials of self-testing within the NHS, though mixed uptake by region. Currently available for commercial purchase for £30-£80. |
| Home blood pressure monitoring during pregnancy | Inconvenience of attending appointments for blood pressure monitoring, concerns about delayed diagnosis | 'blood pressure monitoring', 'pre-eclampsia', 'high blood pressure in pregnancy', 'gestational high blood pressure' AND 'remote monitoring', 'at-home monitoring', 'home monitoring', 'self-monitoring' | [Feasibility study](https://www.sciencedirect.com/science/article/pii/S2210778919304441), [prospective cohort study](https://bmcpregnancychildbirth.biomedcentral.com/articles/10.1186/s12884-017-1605-0), [randomised controlled trial](https://www.sciencedirect.com/science/article/pii/S2210778919304441), and [randomised clinical trial](https://www.sciencedirect.com/science/article/pii/S2210778919304441) of at-home blood pressure monitoring during pregnancy in the UK | Systematic reviews of international evidence on self-monitoring during pregnancy ([1](https://bmcpregnancychildbirth.biomedcentral.com/articles/10.1186/s12884-022-04751-7), [2](https://www.sciencedirect.com/science/article/pii/S2210778919304738)) | 9 | Met | The technology exists and is widely available, however continued work is needed for improved integration into clinical care and producing a robust evidence base |

**Appendix E: Summary of literature searches for needs considered met by existing technologies**

The searches and evidence for the three needs suggested in the information gathering processes which were considered to be adequately met by existing technologies are summarised below in Appendix E, supplementary table 1:

*Appendix E, Supplementary Table 1: the searches and evidence for the three needs considered met by existing technologies*
