# Supplementary material for: What are important areas where better technology would support women’s health? Findings from a priority setting partnership
Source: BMC Womens Health. 2023 Dec 13;23:667. doi: 10.1186/s12905-023-02778-2 (PMC10720144; doi:10.1186/s12905-023-02778-2)
Supplement: Supplementary file 6 — Additional file 6. [file 12905_2023_2778_MOESM6_ESM.docx]

**Appendix F: Unmet needs ten to 20, as identified by the priority setting partnership meeting**

The needs identified in the top 20, from ten to 20 are represented below in supplementary table 4.

Appendix F, supplementary Table 1:

| Wider range/better designs of hormonal contraceptives (including pill formulations, vaginal rings designs and sizes, and patch colours) |
| --- |
| Better pads and products for urine and faecal leakage |
| Better technology to reduce the discomfort of inserting intrauterine contraception (IUC) including self-insertion delivery of pain relief that women could use prior to IUC fitting |
| Better ways to deliver hormones and medication into the vagina to treat dryness and pain due to the menopause  (Merged with – Device for self-insertion of dinoprostone (a medication used to induce labour)) |
| HRT patches which don’t fall off |
| Technologies to support pelvic floor assessment and care – smaller, easier to use than current models, allowing biofeedback and self-visualisation, educating about and supporting relaxation of the pelvic floor when this is causing pain |
| Technology to support recovery from diastasis recti (separation of the abdominal muscles during pregnancy) |
| Tests to help people who struggle to conceive know when they are most fertile |
| Tests to identify menopause better and earlier and learn how to individualise care |
| Ways to visualise your genital anatomy and pelvic floor to help with education and pelvic floor exercises |

*Appendix F, Supplementary Table 1: Unmet needs ten to 20*
